# Effects of Dance-Based Aerobic Training on Mental Health and Quality of Life in Older Adults with Mild Cognitive Impairment

**DOI:** 10.3390/jpm14080844

**Published:** 2024-08-09

**Authors:** Marcelina Sánchez-Alcalá, Agustín Aibar-Almazán, Fidel Hita-Contreras, Yolanda Castellote-Caballero, María del Carmen Carcelén-Fraile, Aday Infante-Guedes, Ana María González-Martín

**Affiliations:** 1 Department of Health Sciences, Faculty of Health Sciences, University of Jaén, 23071 Jaén, Spain; 2 Department of Health Sciences, Faculty of Health Sciences, University of Atlántico Medio, 35017 Las Palmas de Gran Canaria, Spain; aday.infante@pdi.atlanticomedio.es; 3 Department of Education and Psychology, Faculty of Social Sciences, University of Atlántico Medio, 35017 Las Palmas de Gran Canaria, Spain; carmen.carcelen@pdi.atlanticomedio.es (M.d.C.C.-F.);; 4 Department of Psychology, Higher Education Center for Teaching and Educational Research, Plaza de San Martín 4, 28013 Madrid, Spain

**Keywords:** dance-based aerobics, depression, sleep quality, quality of life, older adults, mild cognitive impairment

## Abstract

(1) Background: Mild cognitive impairment in older adults is a condition characterized by a decrease in mental abilities that affects their quality of life. The aim of this study is to evaluate the effects of an aerobic training program based on dance on depression, sleep quality, and quality of life in older adults with mild cognitive impairment. (2) Methods: This study employed a randomized controlled trial design with a total of 92 older adults with cognitive impairment, randomly assigned to an experimental group (*n* = 47) undergoing dance-based aerobic training and a control group (*n* = 45) who did not receive any intervention. Depression was assessed using the Yesavage Geriatric Depression Scale, sleep quality through the Pittsburgh Sleep Quality Index (PSQI), and quality of life through the SF-36 questionnaire. (3) Results: Statistically significant improvements were observed in depression (t(46) = 4.783, *p* = 0.000) and in the PSQI domains: subjective sleep quality (t(46) = 3.333, *p* = 0.002, and Cohen’s d = 0.35), sleep duration (t(46) = 5.511, *p* = 0.000, and Cohen’s d = 0.73) and PSQI total score (t(46) = 2.116, *p* = 0.040, and Cohen’s d = 0.20). Regarding quality of life, improvements were observed in all domains of the questionnaire: the general health (t(46) = −9.374, *p* = 0.000, and Cohen’s d = 0.03), physical function (t(46) = −9.374, *p* = 0.000, and Cohen’s d = 0.03), the physical role (t(46) = −5.954, *p* = 0.000, and Cohen’s d = 1.06), the emotional role (t(46) = −6.200, *p* = 0.000, and Cohen’s d = 0.80), social function (t(46) = −5.585, *p* = 0.000, and Cohen’s d = 0.53), physical pain, (t(46) = −9.224, *p* = 0.000, and Cohen’s d = 1.04), vitality (t(46) = 2.289, *p* = 0.027, and Cohen’s d = 1.27), mental health, (t(46) = −7.985, *p* = 0.000, and Cohen’s d = 1.33), the physical summary component, (t(46) = −13.532, *p* = 0.000, and Cohen’s d = 1.81), and in the mental summary component (t(46) = −10.6 81, *p* = 0.000, and Cohen’s d = 0.06); (4) Conclusions: The results of the present study showed that they suggest that a dance-based aerobic training program improves mental health and quality of life in older people with mild cognitive impairment, providing a non-pharmacological approach to improve general well-being in this population.

## 1. Introduction

The aging of the population is a global trend that poses significant socioeconomic and public health challenges. The global birth rate has experienced a significant decline in recent decades, as it was considerably higher 60–70 years ago due to factors such as lack of access to contraception, cultural and socioeconomic expectations favoring large families, and lower levels of education and employment for women [1]. In contrast, current birth rates are much lower in many parts of the world, due to a combination of improved access to family planning, higher levels of education and female labor force participation, and changing social and economic expectations favoring smaller families [2]. This decline in the birth rate, coupled with advances in medicine and improvements in quality of life, has contributed to population aging [3]. Fewer births combined with increased longevity have resulted in a significant increase in the proportion of older people in the total population [4]. According to the World Health Organization, the proportion of people aged 60 years and older will double from approximately 12% to 22% between 2015 and 2050 [5]. This situation may lead to increased demand for health and long-term care services, putting pressure on health and social welfare systems. It is therefore crucial to address these challenges through policies and programs that support the aging population, promote healthy aging, and ensure long-term socioeconomic sustainability. This demographic shift underscores the need to implement effective strategies to ensure healthy and productive aging.

Older adults often face various health challenges related to mental and physical well-being, such as depression, sleep quality issues, and a general decline in quality of life. One of the most concerning health issues in old age is mild cognitive impairment (MCI), a condition involving a noticeable decline in cognitive abilities without significantly impacting daily activities [6]. MCI can precede more serious neurodegenerative diseases like Alzheimer’s disease and affects a substantial proportion of the elderly population. Symptoms include memory problems, difficulty in planning and executing complex tasks, and challenges with decision-making and critical thinking [7]. It can also be influenced by several nutritional etiologies, as nutrition plays a crucial role in cognitive health, and deficiencies in certain nutrients can contribute to its development and progression [8], such as a lack of vitamin B1 (thiamine) that can contribute to cognitive problems affecting confusion and coordination, vitamin B6 (pyridoxine) deficiency that can interfere with the synthesis of neurotransmitters crucial for cognitive function, and vitamin B12 and folic acid deficiencies that are associated with neurological problems and memory impairment [9]. This mild cognitive impairment not only poses an individual challenge but also has significant implications for the overall quality of life of older adults.

Depression among older adults is a clinical reality affecting approximately 7% of the global population in this age group and is strongly linked to decreased functionality and well-being [10]. Depression in later life not only causes sadness and apathy but can also manifest through somatic symptoms such as chronic pain, fatigue, and sleep disorders, contributing to a significant decline in the affected individuals’ quality of life [11]. Additionally, sleep quality tends to diminish with age, with a large proportion of older adults reporting sleep problems due to changes in sleep patterns, which negatively impact their quality of life [12]. Sleep disorders in older adults, such as insomnia and sleep apnea, are common and can be exacerbated by chronic medical conditions, medication use, and reduced physical activity [13]. The lack of restorative sleep can lead to a vicious cycle where daytime fatigue and sleepiness contribute to reduced participation in social and recreational activities, which are crucial for maintaining overall well-being [14]. Quality of life, a comprehensive indicator of well-being, is affected not only by physical and mental health status but also by the ability to maintain an active and satisfying social life [15]. Older adults who can maintain significant social relationships, participate in community activities, and access social support resources tend to report better quality of life [16]. The perception of control over their environment and the ability to make autonomous decisions also play a crucial role.

Physical exercise has been recognized as a key strategy to improve health and quality of life in older adults. Research has shown that regular physical activity can mitigate the prevalence and severity of depression, improve sleep quality, and overall enhance quality of life in this age group [17,18]. Physical exercise not only improves physical capacity and mental health but also provides opportunities for socialization and community engagement, crucial aspects for perceiving a high quality of life [19]. Regular physical activity contributes to maintaining mobility and independence, which are essential for daily functioning and reducing the risk of falls and fractures [20]. Furthermore, participation in group exercise programs promotes social networking, providing a sense of belonging and emotional support that is vital for psychological well-being [21]. The positive impact of physical exercise on sleep quality has also been studied, highlighting the regulation of the biological clock and a more stable sleep–wake cycle [22]. Studies have shown that aerobic and resistance exercise can reduce the time needed to fall asleep, increase sleep duration, and improve sleep quality [14,23]. This is particularly relevant for older adults, who often face insomnia and other sleep disorders that can negatively affect their overall health and quality of life [24]. Among various forms of exercise, aerobic exercise, including dance, stands out for its multiple benefits. Dance, in particular, is not only an aerobic activity that improves cardiovascular and muscular health but also a social and cultural activity that can significantly enhance the mental and emotional health of participants [25]. Studies have demonstrated that dance can reduce symptoms of depression, improve sleep stability, and overall enhance the quality of life of older adults [26,27].

For all these reasons, the aim of this study was to assess the impact of an aerobic dance-based training program on depression, sleep quality, and overall quality of life in older adults with mild cognitive impairment. Based on the literature reviewed, we hypothesize that aerobic training through dance will not only reduce levels of depression and improve sleep quality in participants but will also improve their overall quality of life. This study hopes to provide robust evidence supporting the use of dance as an effective and accessible intervention to improve the health and well-being of older adults.

## 2. Materials and Methods

### 2.1. Study Design

The design of this research was based on a randomized, controlled clinical study carried out between January and April 2024. Before starting any intervention, all participants were provided with detailed information about this study and gave written consent to take part in this study. This study was approved by the Ethics Commission of the University of Jaén (FEB.23/3.TES), following the ethical guidelines established in the Declaration of Helsinki, and was registered with the number NCT06130878.

### 2.2. Participants

**Figure 1:**
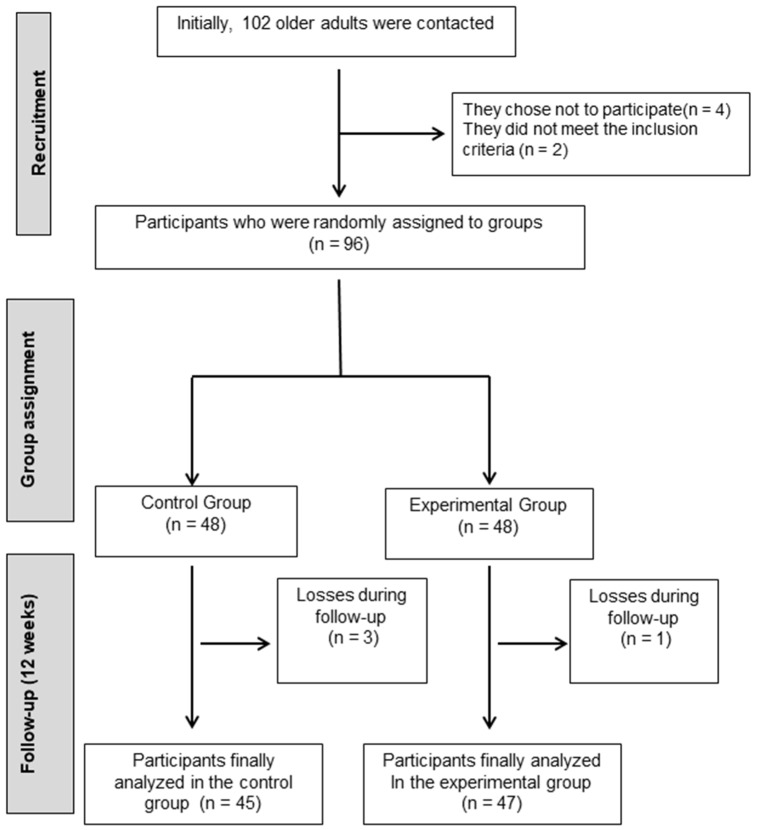
Flowchart of participants in this process.

Initially, contact was established with 102 older adults with mild cognitive impairment. After a selection process, four individuals chose not to participate in this study, and two did not meet the necessary inclusion criteria. Consequently, 96 participants were successfully enrolled and were randomly assigned to two study groups (Figure 1). Older adults who met the following criteria were included in this study: (i) being 65 years or older; (ii) not participating in any physical exercise program; (iii) scoring less than 24 on the MMSE; (iv) having physical autonomy to participate in the physical activities required by this study; (v) understanding the instructions, programs, and protocols of this project; (vi) signing the informed consent form; (vii) completing more than 90% of the exercise intervention. Conversely, older adults were excluded if they (i) had any type of systemic disease (e.g., neurodegenerative, musculoskeletal, or vision-related) that prevented them from performing the postural balance test or exercises; (ii) had any type of vestibular disorder or disease; and (iii) were taking medications that affected the central nervous system, balance, or coordination (e.g., antidepressants, anxiolytics, or vestibular sedatives).

### 2.3. Randomization

The older adults selected to participate in this study were randomly assigned to two groups, one experimental and one control, in a 1:1 ratio. This assignment was carried out using a computer-generated random number table. Group allocation was conducted using sealed opaque envelopes, and this task was carried out by an independent entity that was not involved in participant selection, intervention implementation, or the analysis of the collected variables. A total of 48 participants were assigned to the experimental group (EG), which performed dance-based aerobic training, and 48 participants were assigned to the control group (CG). Those assigned to the control group received specific instructions to maintain their usual daily routines and refrain from participating in any structured training programs. Additionally, they received recommendations to encourage physical activity.

### 2.4. Intervention

The dance-based aerobic training program extended over 12 weeks with a frequency of two sessions per week, totaling 24 sessions. Each session lasted 60 min and was divided into three phases: (A) Warm-up Phase (10 min): This phase included low-intensity activities based on stretching and flexibility movements performed at a slow pace to facilitate adaptation to rhythm and coordination; (B) Core Phase (40 min): During this phase, participants performed moderate-intensity dance steps involving continuous movements of the lower limbs, trunk, and intermittent arm movements. Each song lasted approximately 4 min with 2-min breaks between songs. There was a gradual progression in the complexity of choreographies over the weeks. The dance steps included flexion–extension, abduction and adduction, lateral displacements, rotations, changes in rhythm, forward and backward movements, foot position changes, heel lifts, and movements of the upper and/or lower extremities with a gradual progression in complexity over the weeks. Choreographies were carefully selected and sequenced to ensure a logical progression in technical complexity, and different musical styles (salsa, rock, rumba, pop, merengue, bachata) were used for each choreography; (i) weeks 1–4: The main objective was to familiarize participants with basic steps, introducing choreographies with simple rhythmic patterns and lateral movements, forward and backward steps; (ii) weeks 5–8: Complexity increased with the introduction of new steps and combinations, including turns that required greater coordination and sequential memory; (iii) weeks 9–12: Advanced choreographies were introduced with more complex step combinations aimed at improving retention and recall of complex information through sequences performed without instructor guidance; (C) Cool-down Phase (10 min): This phase focused on gentle stretching exercises, accompanied by relaxing music, to facilitate a transition to a calm state.

This intervention was supervised and led by a qualified instructor with training in physiotherapy and fitness training and previous experience in leading physical exercise programs for older adults. To ensure the safety of participants, the sessions were conducted in suitable and safe facilities, with equipment and spaces designed to minimize the risk of falls or injuries, and clear protocols were established to handle any emergency situation, including the availability of first aid and rapid access to medical services.

### 2.5. Outcomes

An independent researcher who was not involved in assigning participants to the different groups or implementing the intervention was responsible for collecting all data and variables related to this study. Sociodemographic and clinical information was collected, including age, weight (measured from an accurate Tefal digital scale that has a capacity of 100 g to 130 kg), height (measured using an Asimed T201-T4 stadiometer), marital status (married, single, separated/divorced, or widowed), employment status (retired, unemployed, or employed), and educational level (no formal education, primary, secondary, or university).

#### 2.5.1. Depression

The depression variable was evaluated using the Yesavage Geriatric Depression Scale, a validated questionnaire that is in Spanish and designed to detect signs of depression in adults over 65 years of age [28]. In this study, the abbreviated version consisted of 15 questions with a yes/no response, which assessed the emotional state of the individual during the previous week [29]. This scale focuses on the specific cognitive and behavioral aspects of older people related to depression. Its completion takes between 5 and 7 min, granting a maximum possible score of 15 points. An affirmative answer to 10 is considered to indicate the presence of depression, while a negative answer to 5 questions also indicates depression. The scores are interpreted as follows: a score between 0 and 4 is considered normal, between 5 and 8 reflects mild depression, between 9 and 11 indicates moderate depression, and between 12 and 15 indicates severe depression.

#### 2.5.2. Sleep Quality

To analyze sleep quality, the Pittsburgh Sleep Quality Index (PSQI) [30,31] was used, which is recognized as a prominent tool in this area. This questionnaire consists of 19 self-assessment questions and 5 additional questions that must be answered by someone sharing the bed or room with participants (although the latter serve to obtain complementary information). The questions generate a total score and are divided into 7 different components or areas: (i) subjective perception of sleep quality; (ii) time to fall asleep; (iii) duration of effective sleep; (iv) habitual sleep efficiency; (v) interruptions during sleep; (vi) use of sleeping medication; (vii) daytime impact due to sleep problems. The PSQI total score can range from 0 to 21, with a higher score indicating poor sleep quality.

#### 2.5.3. Quality of Life

A quality of life assessment was conducted using the Spanish version of the SF-36 questionnaire (Short Form-36 Health Survey) [32], originally developed by Ware et al. [33]. This tool is one of the most commonly used to measure quality of life, composed of 36 questions that identify both positive and negative aspects of health. These questions are grouped into 8 different areas: physical function (10 questions), physical role (4 questions), bodily pain (2 questions), general health (5 questions), vitality (4 questions), social function (2 questions), emotional role (3 questions), and mental health (5 questions). Additionally, the SF-36 questionnaire provides two summary scores: one for physical health (PCS) and one for mental health (MCS). The total score varies between 0 and 100, where 0 indicates the worst quality of life, while 100 indicates the best.

### 2.6. Sample Calculation

For the depression variable, the sample size was determined based on a 95% confidence level and a statistical power of 90%. An observed effect size (g) of 0.30 was used as reported by Hui et al. [34]. Assuming a similar standard deviation in the population, this resulted in an estimated sample size of at least 31 subjects in each group to ensure the statistical validity of the results. A 20% loss was added to account for attrition, resulting in a total sample size of 74 participants.

### 2.7. Statistical Analysis

All statistical analyses were carried out with the SPSS statistical program, version 20.0 for Windows (SPSS, Inc., Chicago, IL, USA). We used a statistical significance level of *p* < 0.05. The results were presented using means and standard deviations for continuous variables and frequencies and percentages for categorical variables. The Kolmogorov–Smirnov test was used to check the normality of the data distribution. To determine possible differences between the two study groups before the start of this study, Student’s *t*-tests and Chi-square tests were used for continuous and categorical variables, respectively. To analyze differences in values between the studied variables, a mixed analysis of variance was conducted, with the study group as the intergroup factor (CG vs. EG) and the measurement time (pre- and post-intervention) as the intra-group factor. The dependent variables were depression (the Yesavage Geriatric Depression Scale), sleep quality (the Pittsburgh Sleep Quality Index), and quality of life (the Spanish version of the SF-36 questionnaire). All analyses were carried out independently for each dependent variable, and the possible ‘group × measurement time’ interactions were analyzed. To assess the effect size of possible inter-group and intra-group differences, Cohen’s d statistic was used. Values < 0.2 indicate a negligible effect size; values ≥ 0.2 and <0.5 indicate a small effect size; values ≥ 0.5 and <0.8 indicate a medium effect size; and values ≥ 0.8 indicate a large effect size.

## 3. Results

The present study comprised 36.96% men and 63.04% women. The average age of the participants was 71.83 ± 2.96 years. Most of them were retired (45.6%), married (35.4%), and had primary education (36.7%) (Table 1). There were no significant differences in any sociodemographic characteristics between the groups.

### 3.1. Depression 

**Figure 2:**
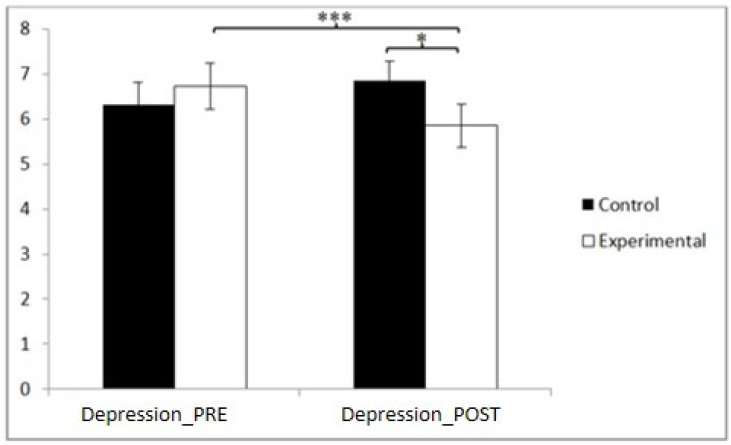
Inter- and intra-group comparisons regarding depression. * *p* < 0.05; *** *p* < 0.001.

According to our findings, in depression, statistically significant differences were found between the pre- and post-measurement in the EG: t(46) = 4.783, *p* = 0.000, and Cohen’s d = 0.13, and statistically significant differences between both groups in the post-intervention measurement: t(90) = 2.205, *p* = 0.030, and Cohen’s d = 0.46 (Table 2 and Figure 2). This indicates that dance-based aerobic training can significantly reduce symptoms of depression in older adults, which is crucial to improving their mental health and quality of life.

Data are expressed as means and standard deviations. Qualitative variables are presented as frequencies and percentages. CG = control group; EG = experimental group; and PSQI = Pittsburgh Sleep Quality Scale.

### 3.2. Sleep Quality

**Figure 3:**
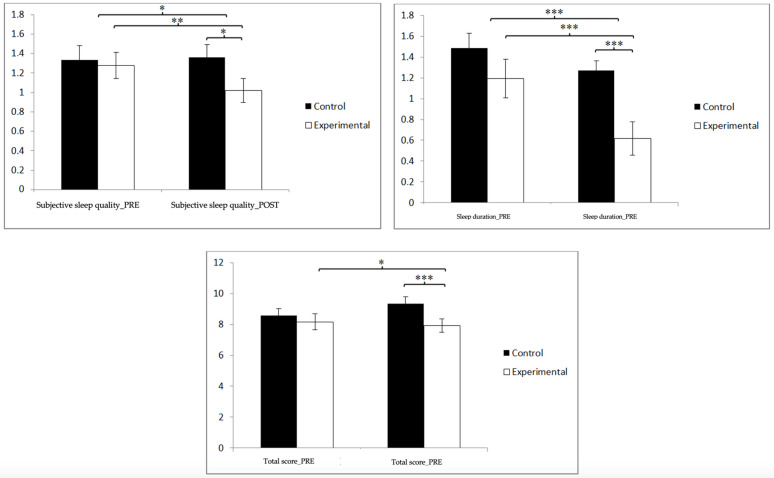
Inter- and intra-group comparisons regarding sleep quality. * *p* < 0.05; ** *p* < 0.01; *** *p* < 0.001.

Regarding sleep quality (Table 2 and Figure 3), in the subjective sleep quality scores, statistically significant differences were found between the pre- and post-measurement in EG: t(46) = 3.333, *p* = 0.002, and Cohen’s d = 0.35, and statistically significant differences between both groups in the post-intervention measure: t(90) = 2.017, *p* = 0.047, and Cohen’s d = 0.42. Regarding sleep duration, statistically significant differences could be observed between the pre- and post-measurements in GE: t(46) = 5.511, *p* = 0.000, and Cohen’s d = 0.73, and statistically significant differences between both groups in the post-intervention measure: t(90) = 2.017, *p* = 0.047, and Cohen’s d = 0.98. Finally, in the PSQI total score, statistically significant differences were observed between the pre- and post-measurement in the EG: t(46) = 2.116, *p* = 0.040, and Cohen’s d = 0.20, and statistically significant differences between both groups in the post-intervention measurement: t(90) = 3.788, *p* = 0.000, and Cohen’s d = 0.79. In contrast, sleep latency, sleep efficiency, sleep disturbances, medication use, and daytime dysfunctions did not show any significant main effect with respect to the group and group × time interaction. These results underline that the training program improves both the perception of sleep quality and its duration, which are fundamental aspects for the general health and daily well-being of the participants.

### 3.3. Quality of Life

**Figure 4:**
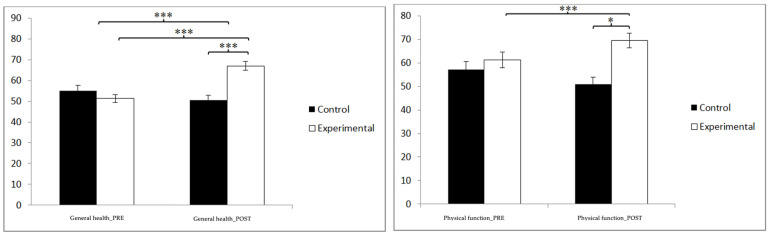
Inter- and intra-group comparisons regarding general health and physical function. * *p* < 0.05; *** *p* < 0.001.

Regarding quality of life (Table 3), statistically significant differences were found in the general health scores between the pre- and post-measurement in the treatment/training group: t(46) = −9.374, *p* = 0.000, and Cohen’s d = 0.03, and statistically significant differences between both groups in the post-intervention measure: t(90) = −5.444, *p* = 0.000, and Cohen’s d = 1.13. In physical function, statistically significant differences were found between the pre- and post-measurement in the treatment/training group: t(46) = −9.374, *p* = 0.000, and Cohen’s d = 0.03, and statistically significant differences between both groups in the measurement. postintervention: t(90) = −4.524, *p* = 0.043, and Cohen’s d = 0.94 (Figure 4). 

**Figure 5:**
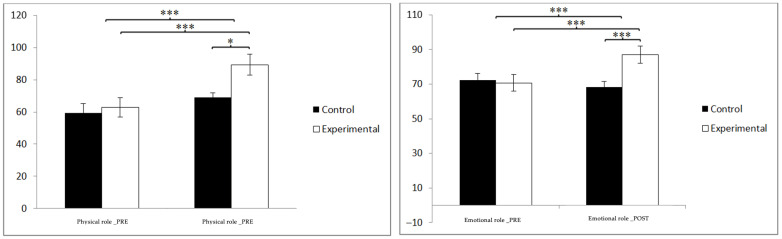
Inter- and intra-group comparisons regarding physical role and emotional role. * *p* < 0.05, *** *p* < 0.001.

In the physical role scores, statistically significant differences were found between the pre- and post-measurement in the treatment/training group: t(46) = −5.954, *p* = 0.000, and Cohen’s d = 1.06, and statistically significant differences between both groups in the post-intervention measure: t(90) = −2.348, *p* = 0.021, and Cohen’s d = 0.49. In the emotional role, statistically significant differences were found between the pre- and post-measurement in the treatment/training group: t(46) = −6.200, *p* = 0.000, and Cohen’s d = 0.80, and statistically significant differences between both groups in the measurement post-intervention: t(90) = −3.348, *p* = 0.000, and Cohen’s d = 0.80 (Figure 5). 

**Figure 6:**
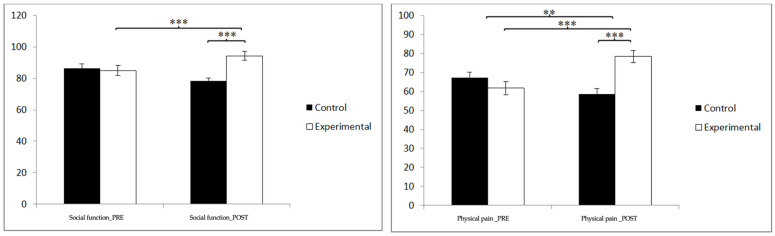
Inter- and intra-group comparisons regarding social function and emotional role. ** *p* < 0.01; *** *p* < 0.001.

In social function, statistically significant differences were found between the pre- and post-measurement in the treatment/training group: t(46) = −5.585, *p* = 0.000, and Cohen’s d = 0.53, and statistically significant differences between both groups in the post-intervention measure: t(90) = −3.668, *p* = 0.000, and Cohen’s d = 0.76. In physical pain, statistically significant differences were found between the pre- and post-measurement in the treatment/training group: t(46) = −9.224, *p* = 0.000, and Cohen’s d = 1.04, and statistically significant differences between both groups in the post-intervention measure: t(90) = 87.028, *p* = 0.000, and Cohen’s d = 0.94 (Figure 6). 

**Figure 7:**
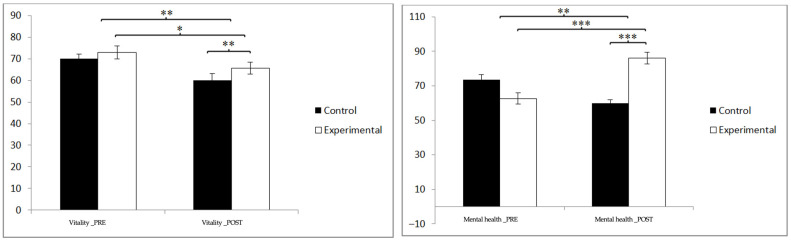
Inter- and intra-group comparisons regarding sleep quality. * *p* < 0.05; ** *p* < 0.01; *** *p* < 0.001.

In vitality, statistically significant differences were found between the pre- and post-measurement in the treatment/training group: t(46) = 2.289, *p* = 0.027, and Cohen’s d = 1.27, and statistically significant differences between both groups in the post-intervention measurement: t(90) = −2.656, *p* = 0.009, and Cohen’s d = 0.55. In mental health, statistically significant differences were found between the pre- and post-measurement in the treatment/training group: t(46) = −7.985, *p* = 0.000, and Cohen’s d = 1.33, and statistically significant differences between both groups in the measurement. postintervention: t(90) = −5.692, *p* = 0.000, and Cohen’s d = 1.18 (Figure 7). 

**Figure 8:**
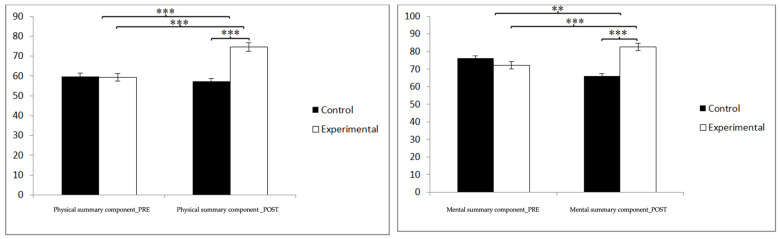
Inter- and intra-group comparisons regarding sleep quality. ** *p* < 0.01; *** *p* < 0.001.

Finally, in the physical summary component, statistically significant differences were found between the pre- and post-measurement in the treatment/training group: t(46) = −13.532, *p* = 0.000, and Cohen’s d = 1.81, and statistically significant differences between both groups in the post-intervention measure: t(90) = −7.806, *p* = 0.000, and Cohen’s d = 1.62. In the mental summary component, statistically significant differences were found between the pre- and post-measurement in the treatment/training group: t(46) = −10.681, *p* = 0.000, and Cohen’s d = 0.06, and statistically significant differences between both groups in the post-intervention measure: t(90) = −5.504, *p* = 0.000, and Cohen’s d = 1.14 (Figure 8). These findings indicate significant improvements across multiple dimensions of quality of life, suggesting that dance-based aerobic training not only benefits specific aspects of physical and mental health but has a broad and holistic positive impact on the well-being of older adults.

Data are expressed as means and standard deviations. Qualitative variables are presented as frequencies and percentages. CG = control group; EG = experimental group; SF-36: The Short Form-36 Health Survey; CSF: physical summation component; and CSM: mental summation component.

## 4. Discussion

This study explored the effects of a dance-based aerobic training program on multiple aspects of health in older adults with mild cognitive impairment, focusing on variables such as depression, sleep quality, and quality of life. The findings showed improvements in depression and in sleep quality, specifically in the subdomains of subjective sleep quality, sleep duration, and the PSQI total score. Regarding quality of life, significant improvements were found in all subscales of the SF-36 questionnaire.

Depression in older adults is a significant public health problem, particularly in those with mild cognitive impairment (MCI), as it can be exacerbated and accelerate the transition to more severe forms of dementia [35]. In our study, significant improvements in depressive symptoms were observed in older adults who participated in a dance-based aerobic training program, with a notable reduction in depression scores both within the experimental group and compared to the control group. These findings are in line with existing literature suggesting that exercise, including dance, may be effective in alleviating symptoms of depression in this population. Blumenthal et al. [36] demonstrated that aerobic exercise can be as effective as antidepressants for the treatment of major depression in older adults without presenting the side effects associated with antidepressants. Similarly, a meta-analysis by Bridle et al. [37] also confirmed the significant effect of exercise in reducing depressive symptoms, while Mura and Carta [38] found that dance improves levels of depression and anxiety, highlighting the additional benefits of the social and recreational component of dance. These results underscore the importance of considering dance as a viable non-pharmacological intervention that offers physical, emotional, and social benefits, suggesting the need for future research to explore different dance styles and frequencies that maximize these therapeutic effects in older adults with MCI.

Quality sleep is essential for physical and mental well-being, especially in older adults, where quality sleep is associated with a better quality of life and a lower prevalence of physical and mental disorders [39]. Sleep disorders are prevalent in older adults, often due to age-related physiological changes, comorbidities, and medications that affect sleep patterns [40]. In this context, aerobic exercise and, specifically, dance, have been studied for their potential to improve sleep quality in this population. Our study evaluated the effects of a dance-based aerobic training program on different aspects of sleep quality in older adults with mild cognitive impairment. Significant improvements were observed in duration and subjective sleep quality, with statistically significant differences both internally in the experimental group and compared to the control group. Specifically, an improvement was reported in sleep duration (t(46) = 5.511, *p* = 0.000, and Cohen’s d = 0.73) and subjective sleep quality (t(46) = 3.333, *p* = 0.002, and Cohen’s d = 0.35). Furthermore, the Pittsburgh Sleep Quality Index (PSQI) total score also showed a significant improvement post-intervention (t(46) = 2.116, *p* = 0.040, and Cohen’s d = 0.20), indicating an overall reduction in sleep problems. Previous studies have investigated the relationship between physical exercise and sleep quality in older adults, often with promising results. Consistent with our results, a study by Reid et al. [41] found that regular aerobic exercise significantly improves sleep quality in older adults with insomnia, potentially through mechanisms related to thermoregulation and stress reduction. Similarly, King et al. [42] demonstrated that structured exercise programs, including walking and light aerobic activities, significantly improved sleep quality in older adults, suggesting that physical activity may be an effective intervention for age-related sleep problems. Additionally, dance, as a form of exercise that combines physical activity and socialization, has shown additional benefits. A study by Kredlow et al. [43] demonstrated that dance may be especially effective in improving sleep quality in older adults due to its pleasant and stimulating nature that can reduce stress and promote a positive mood before sleep.

Quality of life is a comprehensive indicator that reflects the general well-being of an individual, encompassing physical, psychological, and social aspects [44]. In older adults, especially those with MCIs, quality of life is crucial, as increased quality of life can significantly contribute to the treatment of cognitive impairment and improvement in autonomy and life satisfaction [45]. Intervention through physical exercise, especially dance, has proven to be an effective tool to improve quality of life in this demographic group [46]. In our study, significant improvements in multiple aspects of quality of life were observed after participation in a dance-based aerobic training program. Improvements were recorded in general health, physical function, physical and emotional roles, social function, and pain and vitality levels. For example, general health scores showed statistically significant improvements from pre- to post-intervention measurement in the experimental group, with t(46) = −9.374, *p* = 0.000, and Cohen’s d = 0.03. Similarly, social function improved, with a Cohen’s d = 0.53 and a statistically significant difference in the comparison between groups post-intervention (t(90) = −3.668, *p* = 0.000, and Cohen’s d = 0.76). These results suggest that dance not only improves physical aspects but also contributes significantly to social interaction and emotional well-being. Several studies have explored the benefits of physical exercise on the quality of life of older adults with MCI. For example, a study by Venturelli et al. [47] found that aerobic exercise intervention significantly improved health-related quality of life in older adults. Another study by Coubard et al. [48] on dance and its impact on older people demonstrated improvements in coordination, balance, and cognitive function, which indirectly contribute to improving quality of life. Additionally, specific interventions that combine physical exercise with social and recreational activities, such as dance, have been shown to not only improve physical capacity but also reduce social isolation and improve mental health. A study by et al. [49] demonstrated that dance can be a powerful tool to improve the quality of life of older adults by combining physical exercise, music, and socialization.

The generalizability of these findings suggests that the observed benefits of dance-based aerobic training could be extended to other populations of older adults, including those with more severe cognitive impairment. It is possible that individuals with moderate to severe dementia may also experience improvements in depression, sleep quality, and quality of life through tailored exercise programs. Furthermore, the intervention could be culturally tailored to be effective in diverse populations, taking into account the specific musical and dance preferences of each group. Future research should explore the efficacy of dance programs in different cultural contexts and with different severities of cognitive impairment, thus expanding the applicability and potential benefits of this intervention.

Although this study has several strengths, such as the innovative use of dance and a robust controlled design, it also has important limitations. First, only short-term effects were assessed. Longitudinal studies are needed to determine whether the observed benefits are maintained in the long term. Second, this study focused exclusively on older adults with mild cognitive impairment, which limits the generalizability of the findings to populations with more severe cognitive impairment or to older people without cognitive impairment. Furthermore, the nature of this study prevented participants from being blinded to the intervention, which could have introduced biases in the perception and reporting of benefits.

## 5. Conclusions

This study has shown that a dance-based aerobic training program can have significant beneficial effects on depression, sleep quality, and quality of life in older adults with mild cognitive impairment. The results suggest that dance, as a comprehensive physical activity that combines social, emotional, and cognitive elements, offers a promising strategy to improve the overall health of this vulnerable population. Although this study has limitations such as sample size and follow-up duration, the findings point to the potential of dance to be implemented as an accessible and effective intervention in geriatric practice. It is critical that future research expands on these results with larger and more diversified samples and assesses the long-term effects of dance in older adults to further solidify its utility as a therapeutic tool in managing aging and its associated challenges.

## Figures and Tables

**Table 1:** Pre-intervention sociodemographic and clinical characteristics of the participants as a whole and by group.

		Total (n = 92)	EG (n = 47)	CG (n = 45)
Age		71.83 ± 2.96	71.43 ± 2.97	72.24 ± 2.92
Sex	Male	34 (23.10)	18 (52.90)	16 (47.10)
	Female	58 (39.50)	29 (50.00)	29 (50.00)
Occupational status	Retired	67 (45.60)	35 (52.20)	32 (47.80)
	Worker	0 (0.00)	0 (0.00)	0 (0.00)
	Stopped	25 (17.00)	12 (48.00)	13 (52.00)
Civil status	Single	13 (8.80)	7 (53.80)	6 (46.20)
	Married	52 (35.40)	26 (50.00)	26 (50.00)
	Divorced/separated/widowed	27 (18.40)	14 (51.90)	13 (48.10)
Educational status	Without studies	14 (9.50)	8 (57.10)	6 (42.90)
	Primary studies	54 (36.70)	31 (57.40)	23 (42.60)
	Secondary studies	16 (10.90)	5 (31.20)	11 (68.80)
	University studies	8 (5.40)	3 (37.50)	5 (62.50)
Depression		6.46 ± 3.33	6.49 ± 3.30	6.42 ± 3.39
Sleep quality		1.35 ± 0.95	1.34 ± 1.05	1.36 ± 0.86
Sleep latency		1.43 ± 1.12	1.34 ± 1.13	1.53 ± 1.12
Sleep duration		1.20 ± 1.02	1.04 ± 0.96	1.36 ± 1.07
Sleep efficiency		1.11 ± 1.03	0.91 ± 0.95	1.31 ± 1.08
Sleep disturbances		1.58 ± 0.83	1.51 ± 0.75	1.64 ± 0.91
Use of sleeping medication		1.45 ± 1.02	1.51 ± 1.12	1.38 ± 0.91
Daytime dysfunction		1.21 ± 0.73	1.04 ± 0.78	1.39 ± 0.65
PSQI Total score		8.11 ± 3.09	7.66 ± 3.04	8.58 ± 3.11
SF-36 General Health		52.88 ± 15.18	51.60 ± 16.98	54.22 ± 13.10
SF-36 Physical Function		58.32 ± 22.01	60.21 ± 22.21	53.33 ± 21.86
SF-36 Physical Role		60.33 ± 36.72	56.38 ± 37.03	64.44 ± 36.34
SF-36 Emotional Role		71.08 ± 27.49	67.77 ± 27.91	74.53 ± 26.92
SF-36 Social Function		84.40 ± 21.19	82.89 ± 23.43	85.98 ± 18.71
SF-36 Physical pain		63.77 ± 19.26	60.77 ± 17.77	66.91 ± 20.42
SF-36 Vitality		61.09 ± 19.21	52.87 ± 16.38	69.67 ± 18.32
SF-36 Mental Health		68.46 ± 19.22	64.68 ± 19.35	72.40 ± 18.47
SF-36 CSF		58.82 ± 11.59	57.24 ± 12.17	60.48 ± 10.83
SF-36 CSM		73.78 ± 10.82	72.00 ± 10.24	75.64 ± 11.22

Data are expressed as means and standard deviations. Qualitative variables are presented as frequencies and percentages. CG = control group; EG = experimental group; PSQI = Pittsburgh Sleep Quality Scale. SF-36 = Generic quality of life questionnaire; CSF = physical summation component; MSC = mental summation component.

**Table 2:** The effects of a dance-based aerobic training program on depression and sleep quality.

	EG (n = 47)		CG (n = 45)		Group			Time			Group × Time		
	Pre	Post	Pre	Post	F(90)	*p*-Value	η^2^	F(90)	*p*-Value	η^2^	F(90)	*p*-Value	η^2^
Depression	6.49 ± 3.30	5.55 ± 2.78	6.42 ± 3.39	6.93 ± 3.22	1.044	0.310	0.011	1.683	0.198	0.018	19.517	0.000	0.178
Sleep quality	1.34 ± 1.05	1.00 ± 0.91	1.36 ± 0.86	1.36 ± 0.77	1.094	0.298	0.012	6.789	0.010	0.073	6.939	0.010	0.072
Sleep latency	1.34 ± 1.13	1.26 ± 0.97	1.53 ± 1.12	1.60 ± 1.07	1.696	0.196	0.018	0.011	0.916	0.000	0.763	0.385	0.008
Sleep duration	1.04 ± 0.95	0.47 ± 0.55	1.36 ± 1.07	1.18 ± 0.86	9.535	0.003	0096	23.385	0.000	0.206	6.503	0.012	0.067
Sleep efficiency	0.91 ± 0.95	0.70 ± 0.80	1.31 ± 0.08	1.22 ± 0.77	7.290	0.008	0.075	3.163	0.079	0.034	0.533	0.467	0.006
Sleep disturbances	1.51 ± 0.75	1.28 ± 0.68	1.64 ± 0.91	1.51 ± 0.82	1.511	0.222	0.017	7.047	0.009	0.073	0.530	0.469	0.006
Use of sleeping medication	1.51 ± 1.12	1.45 ± 0.12	1.38 ± 0.91	1.47 ± 1.14	0.073	0.787	0.001	0.038	0.846	0.000	1.401	0.240	0.015
Daytime dysfunction	1.04 ± 0.78	0.98 ± 0.79	1.38 ± 0.65	1.13 ± 0.84	3.644	0.059	0.039	2.554	0.114	0.028	0.875	0.352	0.010
PSQI total score	7.66 ± 3.03	7.06 ± 3.05	8.58 ± 3.11	9.29 ± 2.55	7.718	0.007	0.079	0.058	0.811	0.001	7.384	0.008	0.076

**Table 3:** The effects of a dance-based aerobic training program on quality of life.

	EG (n = 47)		CG (n = 45)		Group			Time			Group × Time		
	Pre	Post	Pre	Post	F(93)	*p*-Value	η^2^	F(93)	*p*-Value	η^2^	F(93)	*p*-Value	η^2^
SF-36 General Health	51.60 ± 16.98	67.34 ± 15.28	54.22 ± 13.10	51.11 ± 13.18	5.911	0.017	0.062	24.258	0.000	0.212	54.037	0.000	0.375
SF-36 Physical Function	60.21 ± 22.21	69.89 ± 18.55	56.33 ± 21.86	51.44 ± 20.55	7.252	0.008	0.075	3.395	0.0690	0.036	31.384	0.000	0.259
SF-36 Physical Role	56.38 ± 37.03	88.30 ± 21.40	64.44 ± 36.34	73.33 ± 37.84	0.332	0.566	0.004	30.332	0.000	0.252	9.659	0.003	0.097
SF-36 Emotional Role	67.77 ± 29.91	71.58 ± 28.55	74.53 ± 26.92	90.64 ± 18.22	1.620	0.0206	0.018	18.344	0.000	0.169	30.849	0.000	0.255
SF-36 Social Function	82.89 ± 23.43	81.16 ± 16.70	85.98 ± 18.71	93.45 ± 15.45	1.708	0.195	0.019	2.710	0.103	0.029	19.509	0.000	0.178
SF-36 Physical pain	60.77 ± 17.77	79.09 ± 17.24	66.91 ± 20.42	61.60 ± 19.88	2.789	0.098	0.030	10.787	0.001	0.107	35.597	0.000	0.283
SF-36 Vitality	52.87 ± 16.38	63.67 ± 17.76	69.67 ± 18.32	72.66 ± 14.62	1.899	0.172	0.021	11.133	0.001	0.110	38.945	0.000	0.302
SF-36 Mental Health	64.68 ± 19.35	86.81 ± 13.13	72.40 ± 18.47	65.49 ± 21.89	4.768	0.032	0.050	11.335	0.001	0.112	41.282	0.000	0.314
SF-36 CSF	57.24 ± 12.17	76.15 ± 8.35	60.48 ± 10.83	59.37 ± 12.01	11.690	0.001	0.115	61.859	0.000	0.407	78.173	0.000	0.465
SF-36 CSM	72.00 ± 10.24	83.89 ± 7.99	75.64 ± 11.22	71.34 ± 13.33	5.487	0.021	0.057	9.681	0.002	0.097	44.025	0.000	0.328

## Data Availability

The data presented in this study are available on request from the corresponding author. The data are not publicly available because, due to the sensitive nature of the questions asked in this study, participants were assured raw data would remain confidential and would not be shared.
